# ART, AGING, AND ARTIFICIAL INTELLIGENCE: EXPLORING AGE SCRIPTS IN THE UNIVERSE OF AI-GENERATED ART

**DOI:** 10.1093/geroni/igae098.2986

**Published:** 2024-12-31

**Authors:** Laura Allen, Wenqian Xu, Mariko Nishikitani, Vaishnavi Atul Patil, Sunil Hule, Dana Bradley

**Affiliations:** University of Maryland Baltimore County, Baltimore, Kentucky, United States; Lund University, Lund, Skane Lan, Sweden; University of Maryland Baltimore County, Baltimore, Maryland, United States; University of Maryland Baltimore County, Baltimore, Maryland, United States; University of Maryland Baltimore County, Baltimore, Maryland, United States; University of Maryland Baltimore County, Baltimore, Maryland, United States

## Abstract

This study investigates age-related biases in AI-generated art through a mixed method investigation of images of older and younger individuals. Employing 19 text prompts associated with social exclusion for older adults, we collected 465 images from an AI-art generator, MidJourney, and conducted quantitative and qualitative analyses to understand differences between age group depictions. We used Amazon Rekognition software to detect the numerical image properties. Results that were compared statistically (STATA and Python) revealed significant differences in attributes: images with older people exhibited lower overall brightness (M = 46.54) and sharpness (M = 58.58) compared to younger individuals (brightness: M = 60.50; sharpness: M = 72.55). Moreover, older people were more likely to be smiling (16%) and with eyeglasses (56%) compared to younger individuals (6% and 12%), all with p < 0.001. Next, we conducted a qualitative semiotics analysis with a randomly selected sample of 76 images. Older people were often depicted with a strong emphasis on nostalgia, vulnerability, and aloneness, reflected in vintage-style clothing, worn objects, and worried expressions. In contrast, younger individuals were often portrayed with a focus on modernity, energy, and independence, characterized by vibrant clothing, digital technology, and confident expressions. Together, these findings demonstrate the perpetuation of negative and reductionist age scripts in AI-generated art, such as older adults’ incompetence, loneliness, fear, and outdatedness. Implications of this research extend to societal representations of aging, emphasizing the importance of challenging age-related biases in AI-generated content. Future research should explore refinement of generative models which foster more equitable outcomes.

